# Quality of life and cost-effectiveness of interferon-alpha in malignant melanoma: results from randomised trial

**DOI:** 10.1038/sj.bjc.6602973

**Published:** 2006-01-31

**Authors:** S Dixon, S J Walters, L Turner, B W Hancock

**Affiliations:** 1 Health Economics and Decision Science, and Trent Research and Development Support Unit, School of Health and Related Research, University of Sheffield, Regent Court, 30 Regent Street, Sheffield S1 4DA, UK; 2 Academic Unit of Clinical Oncology, University of Sheffield, Weston Park Hospital, Whitham Road, Sheffield S10 2SJ, UK

**Keywords:** interferon-alpha, adjuvant, melanoma, quality of life, cost-effectiveness

## Abstract

A definitive conclusion regarding the value of low-dose extended duration adjuvant interferon-alpha therapy in the treatment of malignant melanoma is only possible once data on health-related quality of life (HRQoL) and costs have been considered. This trial randomised 674 patients to interferon alpha-2a (3 megaunits three times per week for 2 years or until recurrence) or placebo. Health-related quality of life (QoL) was to be assessed up to 60 months using the European Organisation for Research and Treatment of Cancer (EORTC) QLQ-C30. Data for the economic analysis, including cost information and the EQ-5D were also collected. Patients in the observation (OBS) group had significantly better mean follow-up quality of on five dimensions of the EORTC QLQ-C30 functional scales: role functioning (*P*=0.033), emotional functioning (*P*=0.003), cognitive functioning (*P*=0.001), social functioning (*P*=0.003) and global health status (*P*=0.001). Patients in the OBS group had significantly better mean follow-up symptom scores on seven dimensions of the EORTC QLQ-C30 V1 symptom scales. Economic data showed that costs were £3066 higher in the interferon group and produces an incremental cost per quality-adjusted life year of £41 432 at 5 years. The results show that interferon has significant effects on QoL and symptomatology and is unlikely to be cost-effective in this patient group in the UK.

The role of interferon-alpha in malignant melanoma has long been the debated and researched, with over 6000 patients entered into trials (Molife and Hancock, 2002). One aspect of this has been the effectiveness of low-dose extended duration adjuvant therapy. A recent study in 674 patients with thick primary cutaneous melanoma showed no significant difference in overall survival or recurrence-free survival up to 5 years (Hancock *et al*, 2004). This, together with other trial evidence (Ascierto *et al*, 2006), points to there being no routine role for low-dose therapy within this patient group.

A definitive conclusion is not possible, however, until data on health-related quality of life (HRQoL) and costs have been considered alongside the survival data. It is possible that a HRQoL advantage exists, or that the cost differentials are such that the treatment may be considered cost-effective even in the face of nonsignificant clinical findings.

Data from within AIM-High have been reported on toxicity and change in Karnofsky Performance Status (Hancock *et al*, 2004), but these data offer only a partial view of HRQoL. This paper reports on the HRQoL data from AIM-High plus cost and cost-effectiveness as estimated by an incremental cost per quality-adjusted life year (QALY).

## MATERIALS AND METHODS

Patients within the study were randomised to either interferon alpha-2a at 3 megaunits three times per week for 2 years or until recurrence, or placebo. The study protocol was approved by the relevant research ethics committee and all participating patients gave informed written consent.

HRQoL data in the form of the European Organisation for Research and Treatment of Cancer (EORTC) QLQ-C30 were originally intended to be collected at baseline, 3, 6, 12, 24, 36, 48 and 60 months for a subgroup of patients. However, HRQoL data were actually collected at a variety of time points postrandomisation from 3 days to 77 months. Data for the economic analysis, including cost information and the EQ-5D were collected at 3, 6, 12, 24, 36, 48 and 60 months. These economic data were only collected for a subgroup of patients, selected as every fifth patient to enter the study.

The European Organisation for Research and Treatment of Cancer (EORTC) QLQ-C30 is a 30-item cancer-specific instrument designed to assess the health-related quality of life (QoL) of cancer patients participating in international clinical trials (Aaronson *et al*, 1993). The QLQ-C30 version 1.0 used in the AIM-High Trial incorporated five functional scales: physical (PF), role (RF), cognitive (CF), emotional (EF) and social (SF); three symptom scales: fatigue (FA), pain (PA), nausea and vomiting (NV); a global health status/QoL scale (QL) and six single items assessing additional symptoms commonly reported by cancer patients: dyspnoea (DY), loss of appetite (AP), insomnia (SL), constipation (CO), diarrhoea (DI) and a single item on the perceived financial impact of the disease (FI). All of the scales and single-item measures range in score from 0 to 100. A high scale score represents a higher response level. Thus a high score for a functional scale represents a high/healthy level of functioning; a high score for the global health status/QoL represents a high QoL; but a high score for a symptom scale/item represents a high level of symptomatology/problems.

Patient utilities were obtained from the EQ-5D questionnaire. The EQ-5D is a five-dimensional health state classification. The five dimensions are mobility, self-care, usual activities, pain/discomfort and anxiety/depression. These five dimensions are each assessed by a single question on a three point ordinal scale (no problems, some problems, extreme problems). An EQ-5D ‘health state’ is defined by selecting one level from each dimension. A total of 243 health states are thus defined. Values or preference weights for a sample of these health states were obtained from a general community sample using a Time-Trade-Off (TTO) technique. Estimates for all health states were extrapolated from this sample by statistical regression modelling. The EQ-5D preference-based measure can be regarded as a continuous outcome scored on a −0.59 to 1.00 scale, with 1.00 indicating ‘full health’ and 0 representing dead. The negative EQ-5D scores represent certain health states valued as worse than dead.

Data on resource use covered all key areas of care; interferon dose, inpatient and outpatient hospital care, community nurse and general practitioner care. Data on interferon were collected via the study case report form as completed by the study clinician or research nurse. The other economic data, including the EQ-5D, were collected through a patient completed questionnaire.

### Analysis

Data for the EORTC QLQ-C30 were scored using the EORTC Scoring Manual (Fayers *et al*, 1995). EQ-5D data were scored using UK population values (Dolan, 1997), and combined with mortality data to calculated QALYs (Drummond *et al*, 1997). As baseline EQ-5D values were missing for baseline, these were imputed from a regression of EORTC QLQ-C30 responses on EQ-5D values from other visits.

Differences in patient characteristics between groups were tested for using independent sample *t*-tests, or *χ*^2^ tests, as appropriate. The Kaplan–Meier method was used to calculate the time from randomisation to death, and the log rank test to compare the survival times of both groups (Altman, 1991). HRQoL data were collected at a variety of time points postrandomisation from 3 days to 2136 days, mean 403 days. Patients had between 1 and 13 follow-up QoL assessments, with an average of 3.85 assessments postrandomisation. Given this variation in data collection we decided on a relatively straightforward approach to the analysis of the longitudinal data which involved the use of summary measures (Matthews *et al*, 1990). We summarised follow-up QoL responses for each individual subject by taking the simple average of their follow-up QoL responses over time as our summary measure (Matthews *et al*, 1990) as we were concerned with differences in overall levels of QoL rather than more subtle effects.

Differences in mean follow-up HRQoL between the groups were compared using a multiple linear regression model, with mean follow-up HRQoL as the outcome variable and baseline HRQoL, overall survival status (dead or censored) and treatment group as covariates. *P*-values of less than 0.05 were regarded as being statistically significant.

The economic analysis followed guidelines set down by the National Institute for Clinical Excellence (2004). Costs were calculated by combining resource use data with unit costs representing national estimates (British Medical Association, 2002; Netten and Curtis, 2003). Costs beyond 1 year were discounted at 3.5% per annum. Prices are at 2003/4 levels with prices adjusted using the Hosptial and Community Health Services Pay and Price Index where appropriate (Netten and Curtis, 2003). Cost differences were tested for using independent sample *t*-tests. An incremental cost-effectiveness ratio was calculated using mean costs and QALYs.

Economic data can be severely limited by missing data as both the costs and the QALYs are cumulative measures (i.e. totals over the entire follow-up period). Consequently, if only one value is missing from the full series of follow-up data, the total cannot be calculated. To avoid this problem, missing data imputation becomes an important part of analysing economic data. Within this study, the last observation carried forward was used to impute missing data in order to calculate total costs and QALYs (Heyting *et al*, 1992).

## RESULTS

### Health-related QoL

**Figure 1:**
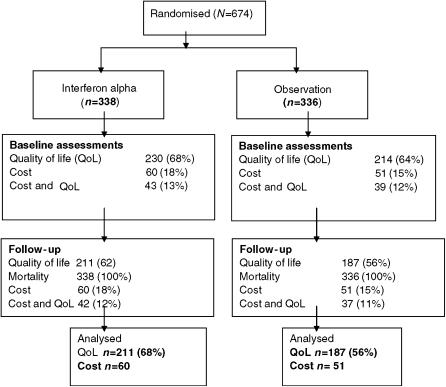
Enrolment, treatment and follow-up of study patients.

Figure 1 shows that 444 patients (out of 674) or 66% had a valid baseline QoL assessment; 230/338 (68%) in the IFN group and 214/336 (64%) in the OBS group (*P*=0.233). Comparison of the *n*=398 patients with a valid baseline QoL assessment and at least one valid follow-up QoL assessment and *n*=276 patients with no baseline or follow-up QoL assessments, suggested that the two groups have similar age (*P*=0.151), gender (*P*=0.349), histology (*P*=0.078), and lengths of follow-up (*P*=0.528) (Table 1).

There was no interaction between treatment group and follow-up QoL assessment status with regard to overall survival (*P*=0.251) and no evidence of a difference in overall survival between the no follow-up QoL data and valid follow-up data groups (log rank *P*=0.84). Median survival was 4.05 years for patients with no valid follow-up QoL data *vs* 3.81 years for patients with valid baseline and follow-up QoL data. This implies we can assume that the QoL sample of 388 patients is a randomly selected subsample of the AIM-High trial population.

**Figure 2:**
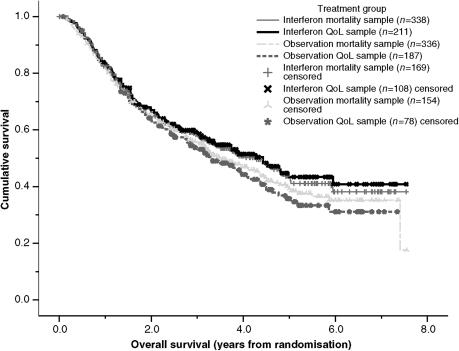
Kaplan–Meier plot of overall survival by treatment group HRQoL follow-up sample.

The IFN and OBS groups in the QoL sample had similar age, gender, stage and overall mortality. The IFN and OBS groups in the QoL sample had similar baseline EORTC QLQ-C30 scores, except for the PAIN dimension, where the IFN had significantly higher levels of pain, +5.8 (95% CI: +1.2 to +10.4, *P*=0.013), see Table 2. There was no evidence of a difference in overall survival between the IFN and OBS groups (log rank *P*=0.15) in the QoL sample. Median survival for IFN was 4.29 years *vs* 3.21 years for the OBS group (see Figure 2).

Patients in the observation (OBS) group had significantly better mean follow-up QoL on five dimensions of the EORTC QLQ-C30 V1 functional scales: RF, EF, CF, SF and QL (see Table 3) after adjustment for baseline QoL and overall survival status (dead or censored). Patients in the OBS group had significantly lower (better) mean follow-up QoL symptom scores on seven dimensions of the EORTC QLQ-C30 V1 symptom scales: FA, NV, DY, AP, CO, DI and FI (see Table 4) after adjustment for baseline QoL and overall survival status (dead or censored).

### Economic evaluation

**Figure 3:**
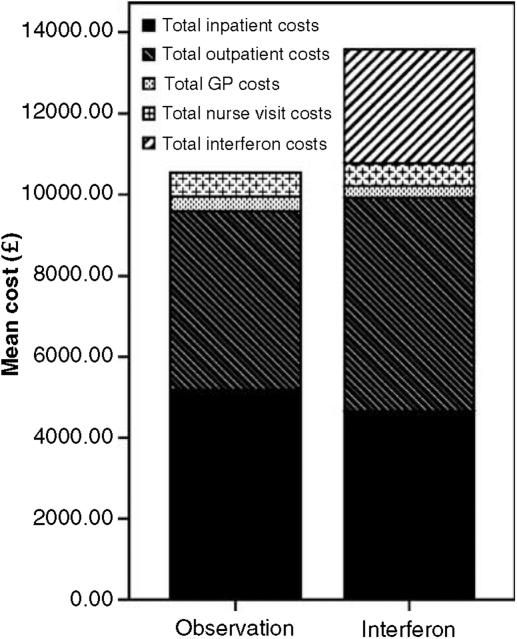
Total costs over 5 years within the two groups (*n*=111).

In total, 134 patients were entered into the economic study and data were available for 111 of these patients. Costs were higher for the interferon (IFN) group in the first 2 years, then slightly lower, thereafter. Overall, costs were £3066 higher in the IFN group. This is almost entirely due to the cost of therapy (Figure 3), but is not statistically significant (*P*=0.396). The IFN group generates 0.074 more QALYs (Table 5), which is equivalent to an extra 27 days in full health, although this is not statistically significant (*P*=0.752).

The incremental cost per QALY for interferon therapy is £41 432. There is considerable statistical uncertainty around this estimate, and a threshold of £30 000 per QALY, there is only a 45% chance of interferon being cost-effective.

## DISCUSSION

These results show that HRQoL is worse in the IFN group in terms of both functioning and symptomatology. As assessed by the EORTC QLQ-C30, statistically significant differences were found in terms of role functioning, emotional functioning, cognitive functioning, social functioning and global health status. Symptom scores in the IFN group were significantly worse for fatigue, nausea/vomiting, dyspnoea, appetite loss, constipation and diarrhoea.

Despite the great interest in interferon therapy for melanoma and its recognised toxicities (Hancock *et al*, 2000), there are very few large scale studies that have used validated HRQoL instruments. Paterson looked at 21 patients receiving high-dose interferon alpha-2b using the Functional Assessment of Cancer Therapy – Biological Response Modifier (FACT-BRM) scale, showing decreased QoL (Paterson *et al*, 2005). In an associated study, Trask looked at 16 patients in a longitudinal analysis which showed reductions in QoL (Trask *et al*, 2004). Bender assessed QoL as part of a trial with 16 patients, and showed a significant reduction in physical well-being associated with high-dose interferon alpha-2b therapy using the Functional Assessment of Cancer Therapy – General (FACT-G) scale (Bender *et al*, 2000).

The largest available study that used a validated QoL measure is by Rataj *et al* (2005) that reported a study of 110 melanoma patients receiving interferon alpha-2b patients following radical surgery. Using the EORTC QLQ-C30 (version 2.0) they found treatment had an impact on physical function, social life, emotional functioning and cognitive functioning. Direct comparisons of that study with this, are not possible due to limited reporting in their paper.

Other work has been undertaken looking at QoL in melanoma patients receiving interferon; however, this has been undertaken with a completely different approach. Kilbridge *et al* (2001), for instance, used the standard gamble technique to value a series of health states describing the QOL associated with interferon toxicity, melanoma recurrence and disease-free health. Their study, based on 107 patient interviews, showed that the side effects from interferon treatment reduced QoL, from 0.96 for the disease-free health state to 0.81 from severe side effects.

The Kilbridge utility estimates have been combined with mortality data from the ECOG *1684* *trial (n*=*280)* to produce a quality-adjusted survival analysis (Kilbridge *et al*, 2002) and a cost-utility analysis (Crott *et al*, 2004). Other analyses have used other utility estimates to describe treatment and post treatment QoL for interferon patients (Cole *et al*, 1996; Hillner *et al*, 1997; Lafuma *et al*, 2001); however, the utility figures were assigned by the researchers rather than derived from patients.

All of these utility-based studies show that a decrease in QoL during interferon treatment is more than offset by improved QoL owing to reduced recurrence and reduced mortality. Consequently, when these utility estimates are combined with the ECOG *1684* data, results tend to show that treatment with high-dose interferon is cost-effective compared to other technologies (Hillner *et al*, 1997; Lafuma *et al*, 2001; Crott *et al*, 2004). These results are in contrast to this study, which shows that while median survival is around 1-year longer, combining QoL with mortality proves the IFN group to be only marginally better (0.074 QALYs, *P*=0.752). This produces an incremental cost-effectiveness ratio of £41 432 per QALY. Using a funding threshold of £30 000 per QALY which is at the higher end of a range used within the United Kingdom (National Institute for Clinical Excellence, 2004), these results show that low-dose extended duration interferon therapy is unlikely to be considered cost-effective.

There are several reasons for these differences. Firstly, AIM-High is a study of low dose interferon therapy, whereas ECOG *1684* is a study of high-dose therapy. Consequently, QoL, survival and recurrence might be expected to differ. Secondly, the utility figures are derived in completely different ways. Our study used a generic preference based outcome measure (EQ-5D) to gather data prospectively from within the trial, from which general population utilities values were applied from a standard algorithm. Kilbridge *et al* (2001) generated utility values from melanoma patients by asking them to value health states describing various treatment scenarios. Thirdly, our study estimates cost-effectiveness at 5 years, while the modelling studies look at longer time scales; 35 years in one case (Crott *et al*, 2004). This is an important difference as shorter time frames generate higher incremental cost-effectiveness ratios. While extrapolation of our results is possible, the lower final year QALY estimate (Table 5) implies that even worse cost-effectiveness results may be produced, if such an analysis were undertaken.

We should also consider the deficiencies associated with our study. Only 66% of patients in the trial had a baseline EORTC QLQ-C30 assessment. Despite this, there appears to be no systematic difference between patients included in our QoL analysis, and those excluded. Another problem was that the number and timing of QoL assessments completed, varied. This led us to undertake a simple analysis, using a summary measure of QoL based on the average scores. As assessments were more frequent during interferon treatment in order to capture the impact of side effects, the results will be weighted toward the early months of treatment. However, repeating the analysis with average follow-up over the first 2 years as the outcome, rather than the total follow-up gave almost identical results to the longer follow-up (data not shown).

We followed the advice of Cox *et al* (1992) for QoL studies which recommended simplicity of design, analysis and presentation of QoL assessments. Therefore, we decided to use a simple approach and not the simultaneous assessment of QoL and survival. There are several approaches to the simultaneous assessment of QoL and survival including: QALYs (for which we employed the EQ-5D), Q-TWiST (quality-adjusted time with spent with symptoms of disease and toxicity of treatment) and multistate survival analysis (Billingham *et al*, 1999). The latter two approaches would require the definition of a finite number of health states in terms of the 15 EORTC QLC-30 dimension scores. We felt it was very difficult to define a set of finite, mutually exclusive and exhaustive health states that are clinically meaningful and fully describe the experiences of patients with malignant melanoma using the 15 dimensions of the EORTC QLC-30.

We assumed that the missing QoL data are missing at random and that dropout was noninformative. We found that the dropout rates and survival experience were similar across the treatment arms and believe that the between-treatment comparisons of QoL remain unbiased. We also included a term for overall survival status in our regression model to adjust for whether the patient was alive or dead during follow-up. This term should take into account that patients who died during follow-up may have a different average QoL at follow-up than patients who were alive or censored.

Further loss of data was present when the economic results are considered, such that data on only 111 patients were available for analysis. Even for these patients, missing data meant that imputation was required to produce a rectangular data set. While differences between this economic subsample and the full sample are not statistically significant, we are limited in our ability to detect differences between the two arms due to the smaller sample size. This problem is perhaps compounded by the possible insensitivity of the EQ-5D seen in several studies (Harper *et al*, 1997; Nicholl *et al*, 2001; Patel *et al*, 2004). Taken together, the lack of a clear pattern in the QALY estimates shown in Table 5 is difficult to interpret.

Looking beyond this study, it is difficult to cast light on other QoL evidence and economic evaluations, as methods are different, as too are the interferon dosing regimens. However, given that such a clear picture of QoL is produced with the EORTC QLQ-C30 we would recommend its use for further studies of interferon treatment. The much cited ECOG 1684 study did not incorporate prospective QoL assessment, and so subsequent evaluations have had to add on supplementary studies. While several improvements to future economic evaluations have been suggested (Crott, 2004), basing future evaluations on trial-based QoL and/or utility estimates would appear to be important given the differences identified here.

Few studies have assessed the impact of interferon therapy on health related QoL using validated instruments. These results show that interferon has significant effects on QoL and symptomatology. Our associated economic analysis also showed that overall, adjuvant low-dose extended duration interferon therapy does not appear cost-effective in this patient group in the UK context.

## Figures and Tables

**Table 1:** Comparison of samples analysed

	**Economic sample (*n*=111)**		**HRQoL sample (*n*=444)**		**HRQoL follow-up sample (*n*=398)**		**Full sample (*n*=674)**	
	***n***	**%**	***n***	**%**	***n***	**%**	***n***	**%**
Overall survival status								
Censored	58	52.3	202	45.5	186	46.7	323	47.9
Died	53	47.7	242	54.5	212	53.3	351	52.1
Total	111	100.0	444	100.0	398	100.0	674	100.0
								
Gender								
Female	42	37.8	186	41.9	166	41.7	292	43.3
Male	69	62.2	258	58.1	232	58.3	382	56.7
Total	111	100.0	444	100.0	398	100.0	674	100.0
								
Stage								
L	6	5.4	41	9.2	35	8.8	74	11.0
LM	23	20.7	89	20.0	77	19.3	130	19.3
RMD	16	14.4	53	11.9	46	11.6	85	12.6
RMR	66	59.5	261	58.8	240	60.3	385	57.1
Total	111	100.0	444	100.0	398	100.0	674	100.0
								
Age								
Mean	51.59			51.6	51.4			52.0
(s.d.)	13.29			12.9	12.7			13.1

L=localised; LM=locally metastatic; RMD=regionally metastatic at diagnosis; RMR=regionally metastatic at recurrence.

**Table 2:** Baseline clinical characteristics and QoL in control and intervention (*n*=398)

	**Group randomised to**					
	**Interferon**			**Observation**		
	***n***	**Mean**	**s.d.**	***n***	**Mean**	**s.d.**
Patient characteristics						
Age (years)	211	50.6	(12.6)	187	52.3	(12.8)
Time (days) between entry to study and first QoL assessment	211	1.1	(2.8)	187	1.0	(1.6)
Time (days) between first and last QoL assessment	211	392.2	(353.3)	187	417.0	(342.3)
No. of valid follow-up QoL assessments	211	3.8	(2.3)	187	3.9	(2.4)
EORTC QLQ-C30 function domains						
Physical functioning	211	88.7	(19.8)	187	89.0	(19.4)
Role functioning	210	83.1	(29.5)	187	84.2	(27.0)
Emotional functioning	211	79.1	(20.7)	187	79.7	(21.1)
Cognitive functioning	211	89.5	(19.1)	187	91.4	(15.8)
Social functioning	211	80.2	(26.3)	187	82.0	(25.3)
Global health status/Qol	211	71.4	(20.9)	187	73.8	(19.3)
EORTC QLQ-C30 symptom scores						
Fatigue	211	21.7	(23.3)	187	18.5	(21.3)
Nausea and vomiting	211	2.4	(6.9)	187	2.0	(8.6)
Pain	211	19.8	(25.3)	187	14.0	(20.7)
Dyspnoea	211	5.1	(12.4)	187	6.6	(15.4)
Insomnia	211	21.6	(25.8)	187	20.1	(29.6)
Appetite loss	210	4.6	(14.8)	187	4.8	(15.3)
Constipation	211	6.5	(17.7)	187	5.2	(14.8)
Diarrhoea	211	3.6	(12.7)	187	3.6	(10.9)
Financial difficulties	211	15.0	(27.2)	187	14.3	(28.7)

For the EORTC QLQ-C30 v1 function scales a higher score represents a better level of functioning.

For the EORTC QLQ-C30 v1 symptom scales a higher score represents a worse level of symptoms.

**Table 3:** Baseline and follow-up EORTC-QLQ-C30 v1 function scores by group (*n*=398)

	**Baseline**			**Follow-up**			**Adjusted difference**	
**Group**	***n***	**Mean**	**s.d.**	**Mean**	**s.d.**	**Difference**	**(95% CI)**	***P*-value**
Physical functioning								
IFN	211	88.7	(19.8)	85.9	(20.2)	2.2	2.1	0.144
OBS	187	89.0	(19.4)	88.1	(16.8)		(−0.7 to 4.5)	
								
Role functioning								
IFN	210	83.1	(29.5)	80.8	(26.2)	5.1	4.3	0.033
OBS	187	84.2	(27.0)	85.6	(22.1)		(0.4–8.3)	
								
Emotional functioning								
IFN	210	79.0	(20.7)	79.4	(19.0)	4.7	4.5	0.003
OBS	187	79.7	(21.1)	84.1	(17.5)		(1.6–7.4)	
								
Cognitive functioning								
IFN	211	89.5	(19.1)	87.3	(16.6)	5.1	4.1	0.001
OBS	187	91.4	(15.8)	92.5	(12.6)		(1.8–6.4)	
								
Social functioning								
IFN	210	80.1	(26.4)	84.9	(19.3)	5.0	4.4	0.003
OBS	187	82.0	(25.3)	89.9	(17.0)		(1.5–7.3)	
								
Global health status/Qol								
IFN	210	71.6	(20.8)	67.5	(19.2)	7.2	5.9	0.001
OBS	187	73.8	(19.3)	74.7	(16.5)		(3.1–8.7)	

For the EORTC QLQ-C30 v1 function scales a higher score represents a better level of functioning.

The treatment group difference in mean follow-up scores is adjusted for baseline score and overall survival status (dead or censored).

A positive follow-up difference implies the observation (OBS) group has a better level of functioning at follow-up, than the interferon (IFN) group.

**Table 4:** Baseline and mean follow-up EORTC-QLQ-C30 v1 Symptom scores by group (n=398)

	**Baseline**			**Follow-up**		**Follow-up**	**Adjusted difference**	
	***n***	**Mean**	**s.d.**	**Mean**	**s.d.**	**Difference**	**(95% CI)**	***P*-value**
Fatigue								
IFN	211	21.7	(23.3)	29.0	(21.7)	−10.8	−9.1	0.001
OBS	187	18.5	(21.3)	18.2	(17.7)		(−12.1 to 6.1)	
								
Nausea and vomiting								
IFN	211	2.4	(6.9)	6.1	(10.8)	−3.4	−3.4	0.001
OBS	187	2.0	(8.6)	2.7	(8.6)		(−5.3 to −1.6)	
								
Pain								
IFN	211	19.8	(25.3)	16.8	(21.1)	−2.7	−0.1	0.937
OBS	187	14.0	(20.7)	14.0	(18.2)		(−3.4 to 3.1)	
								
Dyspnoea								
IFN	211	5.1	(12.4)	12.2	(16.2)	−2.7	−3.7	0.010
OBS	187	6.6	(15.4)	9.5	(16.8)		(−6.5 to −0.9)	
								
Insomnia								
IFN	211	21.6	(25.8)	23.1	(23.6)	−3.8	−3.2	0.123
OBS	187	20.1	(29.6)	19.4	(23.4)		(−7.2 to 0.9)	
								
Appetite loss								
IFN	210	4.6	(14.8)	12.4	(20.8)	−5.8	−6.2	0.001
OBS	187	4.8	(15.3)	6.6	(15.1)		(−9.5 to −3.0)	
								
Constipation								
IFN	211	6.5	(17.7)	7.9	(16.6)	−3.7	−3.3	0.011
OBS	187	5.2	(14.8)	4.1	(10.8)		(−5.8 to −0.7)	
								
Diarrhoea								
IFN	211	3.6	(12.7)	8.8	(13.6)	−4.5	−4.4	0.001
OBS	187	3.6	(10.9)	4.3	(11.8)		(−6.9 to −2.0)	
								
Financial difficulties								
IFN	210	15.1	(27.3)	11.8	(23.6)	−4.8	−4.7	0.001
OBS	187	14.3	(28.7)	7.0	(15.6)		(−7.4 to −2.0)	

For the EORTC QLQ-C30 v1 symptom scales a higher score represents a worse level of symptoms.

The treatment group difference in mean follow-up scores is adjusted for baseline score and overall survival status (dead or censored). A negative follow-up difference implies the observation (OBS) group has a lower/better level of symptoms, at follow-up, than the interferon (IFN) group.

**Table 5:** Profile of quality-adjusted life years for interferon and control patients

	**Mean QALYs (s.d.)**					
	**Year 1**	**Year 2**	**Year 3**	**Year 4**	**Year 5**	**Total**
Observation group (*n*=51)	0.79 (0.18)	0.62 (0.27)	0.43 (0.37)	0.29 (0.35)	0.19 (0.31)	2.33 (1.25)
Interferon group (*n*=60)	0.76 (0.22)	0.67 (0.29)	0.50 (0.34)	0.31 (0.35)	0.16 (0.29)	2.40 (1.19)

Quality-adjusted life years are calculated by multiplying quality of life by length of life, such that 1 year in full health is equivalent to one quality-adjusted life year (QALY). When 1 year produces less than one QALY, this reflects less than full health, for example, 0.5 QALYs is 1 year in a health state valued at 0.5, which is deemed to be equivalent to 6 months (0.5 years) in full health.
